# Proprioceptive training methods (PTM) in female soccer players – a systematic review

**DOI:** 10.1186/s13102-024-00892-8

**Published:** 2024-04-30

**Authors:** Mima Stanković, Ilma Čaprić, Borko Katanić, Omer Špirtović, Džejla Maljanović, Hamza Nailović, Iso Muković, Igor Jelaska, Nebojša Trajković

**Affiliations:** 1 Faculty of Sport and Physical Education, University of Niš, Čarnojevićeva 10a, Niš, 18000 Serbia; 2 Faculty of Sport and Physical Education, State University of Novi Pazar, Novi Pazar, 36300 Serbia; 3 Faculty of Kinesiology, University of Split, Split, 21000 Croatia

**Keywords:** Proprioceptive exercise, Football, Performance, Injury prevention

## Abstract

**Background:**

Although previous studies have reported that proprioceptive training methods (PTM) have positive effects, there is a relatively small number of studies regarding the impact of PTM in women’s soccer. Therefore, there is a need to systematize the given results. In this regard, this systematic review aimed to investigate the effect of proprioceptive training methods in female soccer players.

**Methods:**

The studies’ search and analysis were done according to the PRISMA guidelines. The following databases were checked (Google Scholar, PubMed Cochrane and ProQuest), with additional publication time criteria (2000–2023) using the following keywords: proprioceptive, balance, neuromuscular, training, exercise, intervention, method, activity, female football players, female soccer players, woman soccer players.

**Results:**

A total of 7 studies were included in the quantitative synthesis that meet all the criteria with the number of participants being 2.247. Based on the analysis of the previous research and detailed discussion, the main findings of the study resulted in the partial improvement of explosive strength (66%), strength (50%), muscle imbalance and flexibility (50%) and the prevention and reduction of lower extremity injuries in female soccer players (60%). Only one study reported no significant differences between groups, where rate of major injuries was higher in experimental group.

**Conclusion:**

The obtained results indicate the necessity to implement proprioceptive training in female soccer training programs, in order to influence the prevention and reduction of injuries and improve balance, proprioceptive ability and body control.

## Key points

- PMT can be used in women’s soccer as an excellent weekly training strategy.

- PTM reduces lower extremity injuries and improves explosive strength, strength, agility and muscle imbalance in female soccer players;

- PMT should be an essential part of training programs to prevent injuries and improve athlete’s performance.

## Introduction

Proprioception is the sensitivity of the central nervous system (CNS) to the information it receives from bones, joints, and muscles regarding their position and muscle tone [1]. It is a complex neuromuscular process that deals with kinesthetic awareness of body position and plays an important role in joint stability and injury prevention [2–4]. The basic principle on which proprioceptive training methods (PTM) are based is the stimulation of maintaining balance in different positions or during different movements [2]. The goal of PTM is to enhance the kinesthetic sense of the body position and body parts in space, increase the amplitude of movement in the joints, improve balance, and strengthen the ligamentous-tendon apparatus [5, 6]. The programs focused on restoring balance and proprioception have also been referred to as sensorimotor and neuromuscular training [7]. PTM is frequently used in prevention and rehabilitation through exercises such as balancing on a board with eyes open or closed, throwing and catching a ball while standing on one leg, and so on [2, 4].

Soccer requires various skills such as speed, coordination, endurance, and agility at varying intensities, as well as a wide range of motor movements including jumps, space disputes, and changes of direction. As a result of all of these demands, injuries happen throughout the entire season due to physical contact, sprains and muscle overload [8]. Understanding the intricate relationship among neuromuscular control and training load is critical for identifying players at high risk of injury and developing effective preventative techniques [9]. Previous research has shown that PTM can be effective in improving ankle stability, reducing ankle injuries [10, 11], and enhancing joint position sense, postural sway, and dynamic neuromuscular control [12]. For example, a study conducted by Hübscher et al. [13] found that PTM resulted in a 39% reduction in lower extremity injuries and a 50% reduction in ankle sprain injuries. In soccer specifically, there is a systematic review that shows that PTM increases the proprioceptive ability and improves body control of professional soccer players [14]. Additionally, PTM has been found to be effective in preventing lower extremity injuries in soccer players, according to a study by Ojeda, Sandoval & Barahona-Fuentes [15]. Furthermore, the development of PTM can affect technical skills such as juggling, shooting, heading, passing, and dribbling, showing an overall improvement in neuromuscular coordination, proprioception, and balancing capacities [16].

However, it is worth mentioning that most of these studies have focused on male soccer players, and there is a lack of research on the effects of PTM in female soccer players. Although there are some individual studies on the impact of PTM in women’s soccer, the results are not consistent, which highlights the need for a systematic review to synthesize the existing research and provide a more comprehensive understanding of the effectiveness of PTM in female soccer players. Therefore, the aim of this study is to conduct a systematic review to investigate the effect of proprioceptive training methods in female soccer players. This study is expected to contribute to the knowledge base on PTM in female athletes and inform the development of effective injury prevention and rehabilitation programs.

## Methods

### Literature identification

This study was conducted in regard to the PRISMA guidelines [17, 18]. In order to realize the literature identification, multiple databases (Google Scholar, PubMed Cochrane and ProQuest), with additional publication time criteria (2000–2023) were taken into consideration. To identify the relevant studies that have reported proprioceptive training methods in female soccer players, in mentioned databases were used the combinations of the following keywords: („proprioceptive“ OR „balance“ OR „neuromuscular“ OR „vestibular sense“ OR „stability“ OR „equilibrium“ OR „stabilization“) AND („training“ OR „exercise“ OR „intervention“ OR „method“ OR „activity“) AND („female football players“ OR „female soccer players“ OR „female footballers“ OR „woman soccer players“).

After the initial identification, study evaluations were conducted. A descriptive method was used and all titles, abstracts, and full-text articles were reviewed for possible inclusion. Two authors were independently conducting a study search and evaluation, with the references list from previously assessed studies. Then, each author cross-examined the all identified literature and from that search point, studies were included for further analysis or excluded.

### Inclusion criteria

Eligibility criteria were presented according to the PICO model for eligibility criteria (participant, intervention, comparison, and outcome) [19]. In order for the study to be included in the final analysis, it was necessary that the study satisfies the following inclusion criteria: a full-text study in English, that have published between 2000 and 2023, the experimental studies that have included healthy female soccer players as participant sample, and that participants were tested for their balance or proprioceptive abilities. Exclusion criteria were studies that have had injured participants. In addition, studies where proprioceptive training was used in a manner of injury recovery were not taken for further analysis.

### Risk of bias assessment

The PEDro scale was used to assess the potential bias risk [20]. Two authors (B.K. and M.S.) were independently conducting the mentioned, whereas the authors’ concordance was estimated using k-statistics data. If a disagreement happened, the competent third author (I.C.) was giving the final decision. The k rate of concordance between reviewers’ findings was k = 0.96.

### Data extraction

The relevant information was retrieved only after cross-examination and if the data was suitable with the study’s aim. Following, the Cochrane Consumer and Communication Review Group’s [21] was used to extract the information, such as first author and year of publication, sample size and age, study duration, program type, intensity, frequency with training duration, along with the study outcomes and results.

## Results

### Quality of the studies

Based on the points each study received on the PEDro scale, the final study evaluation results were defined. According to Maher et al. [22] a score between 0 and 3 points will classify that study as “poor” quality, 4–5 points as “fair” quality, 6–8 points as “good” and 9–10 points as “excellent” quality. Of all studies included in this systematic review, 4 studies showed fair quality, and 3 studies showed good quality, which is shown in Table 1. The average score of all studies is 5.3, which means fair quality.

**Table 1:** Pedro scale results

	Criterion											
Study	1	2	3	4	5	6	7	8	9	10	11	∑
Soderman et al. [20]	Y	Y	*N*	Y	*N*	*N*	*N*	Y	Y	Y	Y	6
Knobloch et al. [21]	Y	*N*	*N*	*N*	*N*	*N*	*N*	Y	Y	Y	Y	4
Soligard et al. [22]	Y	Y	*N*	Y	*N*	Y	Y	Y	Y	Y	Y	8
Kraemer et al. [23]	Y	*N*	*N*	*N*	*N*	*N*	*N*	Y	Y	Y	Y	4
Gidu [24]	Y	*N*	*N*	Y	*N*	*N*	*N*	Y	Y	Y	Y	5
Rodriguez et al. [25]	Y	*N*	*N*	*N*	*N*	*N*	*N*	Y	Y	Y	Y	4
Souglis et al. [26]	Y	Y	*N*	Y	*N*	*N*	*N*	Y	Y	Y	Y	6

Legend: 1—eligibility criteria; 2—random allocation; 3—concealed allocation; 4—baseline comparability; 5—blind subject; 6—blind clinician; 7—blind assessor; 8—adequate follow-up; 9—intention-to-treat analysis; 10—statistical analysis; 11—point estimates and variability; Y—criterion is satisfied; N—criterion is not satisfied; ∑—total awarded points

**Fig. 1:**
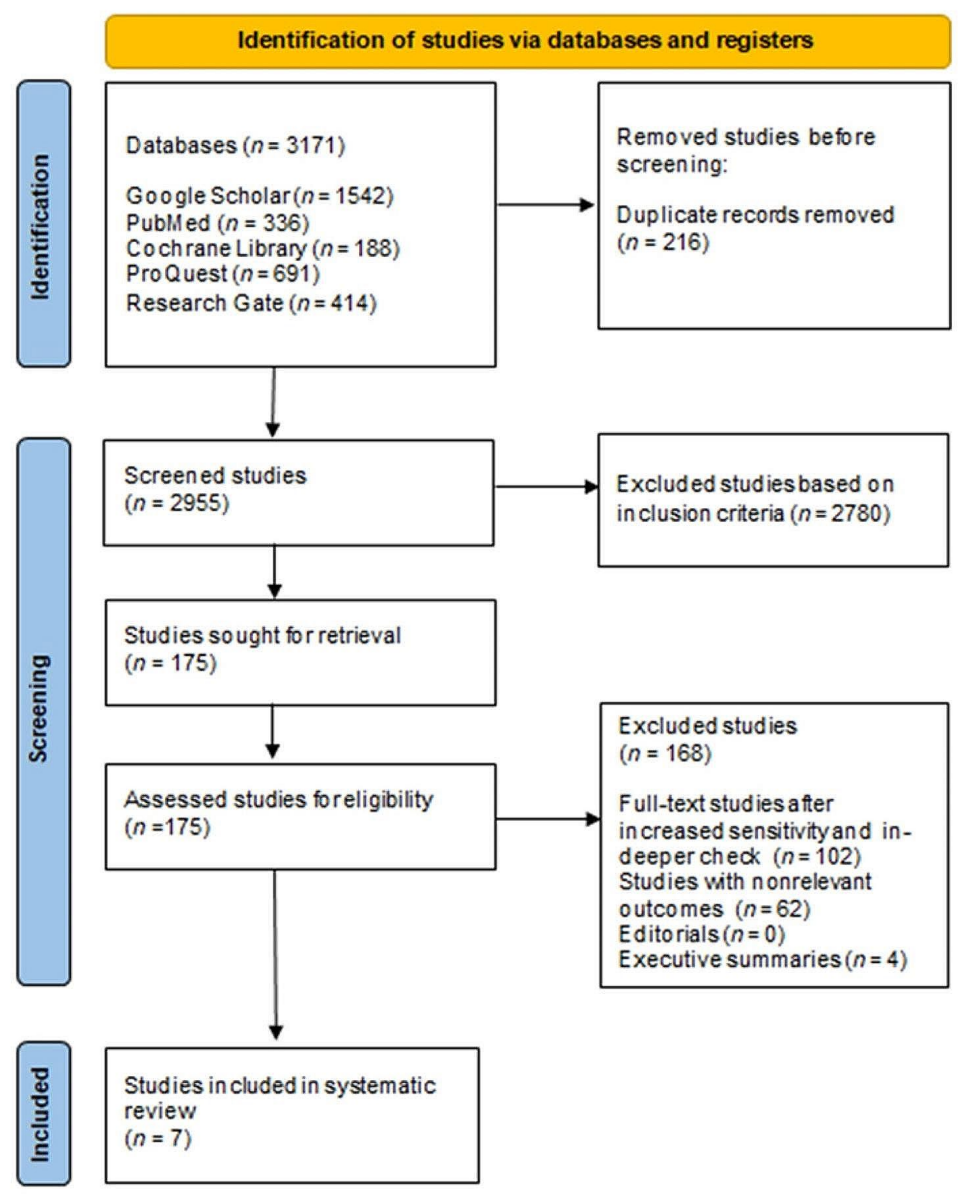
Process of identifying studies for the systematic review

### Study selection and characteristics

A search of electronic databases and a review of study reference lists yielded a sum of 3171 studies. Following an assessment of the duplicates, 216 studies were excluded. After removing the duplicates, the inclusion criteria were analyzed and only 175 studies were considered eligible, with 168 of them being further rejected due to in-depth checks, non-relevant results, editorials, and executive summaries. Finally, seven full-text studies were considered appropriate for the systematic review (Fig. 1).

Furthermore, eligible studies that have included in the systematic review were also, more precisely, presented in Table 2.

**Table 2:** Studies included in the qualitative analysis

First author and year of publication	Participants		Duration (weeks)	Program (type, intensity frequency, training duration	Measured outcomes	Results	
	Age (Years)	Number and groups				E	C
Soderman et al. (2000) [23]	20.5 ± 5.4	E-121 C-100	12	10–15 min of training on a balance board, five exercises with progressively increasing degree of difficulty, 3 × 15 s on each leg, 3 × week	ACL MCL LCL	ACL↔ MCL↔ LCL ↔	ACL↔ MCL↔ LCL ↔
Knobloch et al. (2005) [24]	19–23	E-24	All season	proprioceptive training; Balance-Board-Training	LLG RLS	LLG↑ RLS↑	
Soligard et al. (2008) [25]	13–17	C-837 E-1055	8 months	FIFA 11 + program including three levels of balance exercises in single-leg stance: L.1 hold the ball, L.2 throwing the ball with a partner and L.3 test your partner 3 × week	Injury (foot, ankle, lower leg, knee, thigh, groin, hip)	E group showed significantly lower risk of injuries overall (0.68, 0.48 to 0.98), overuse injuries (0.47, 0.26 to 0.85), and severe injuries (0.55, 0.36 to 0.83).	The risk of injury was 35% higher in C group.
Kraemer et al. (2009) [26]	21 ± 4	E-24	3 years	“protective balancing” principles, 15 and 30 s, 1 × week		Hamstring, (43%) gastrocnemius strains (38%) and back muscle (43%) injuries reduction. There was no effect on contact injuries.	
Gidu (2016) [27]	20.7 ± 4.5	18	6	15 min of proprioceptive exercises, 2 × week	Hop test- consists of five kinds of jumping	S1↔ S2↑ S3↑ S4↑ S5↑	S1↔ S2↔ S3↔ S4↔ S5↔
Rodriguez et al. (2018) [28]	18.6 ± 2.7	E-20	24	PEP program, 3 × week, 20 min	Vertical jump Muscle strength Knee valgus alignment	RQ↑* LQ↑* RH↑ LH↑	
Souglis et al. (2022) [16]	26.9 ± 2.9	E-24 C-24	16	PTIP, 20 min, 5 × week	VO_2_ max JT ST PT SD	VO_2_ max ↑* JT↑* ST↑* PT↑* SD↑*	VO_2_ max ↑ JT↔ ST↔ PT↔ SD↔

Note: N-total number of participants; E-experimental group; E-experimental group ; 2; C-control group; ↑-statistical significance (*p* < 0.05); ↑*-statistical significance (*p* < 0.01); ↓-statistical decrease (*p* < 0.05); ↓*-statistical decrease (*p* < 0.01); ↔-maintaining results; S1 – vertical jump; S2 – hop for distance; S3 - drop jump followed by a double hop for distance; S4 - square hop; S5 - side hop.; RQ - Right quadriceps; LQ - Left quadriceps; RH - Right hamstrings; LH - Left hamstrings; PTIP - Proprioceptive Training Intervention Program; JT - Juggling tests; ST - Shooting test; PT - Passing tests; SD - Speed dribbling; Star Excursion Balance Test (SEBT); ankle dorsiflexion (ADF) and plantarflexion (APF) ; leg extension (LE) and flexion (LF) hip extension (HE) and flexion (HF); ACL anterior cruciate ligament injury, MCL medial collateral ligament injury, LCL lateral collateral ligament injury); PEP- prevent injury and enhance performance; LLG- coordination on the left leg gyroscope; RLS-coordination right leg on the spinning

All studies that met the inclusion criteria were original scientific studies published in English between 2000 and April 2023. The total number of samples was 2247 female subjects, where the largest number of subjects was in the study of Soligard et al. [25] and the smallest in the studies of Gidu [27] with 18 participants and Rodriguez et al. [28] 20 participants. The age of the participants ranged from 13 to 26 years. The longest third process lasted 3 years [26], and the shortest 6 months [27]. Prevent injuries and improve performance and proprioceptive training was the most effective. The majority of the studies revealed significant differences between the groups after the interventions Knobloch et al. [24], Soligard et al. [25], Kraemer et al. [26], Gidu [27], Rodriguez et al. [28], Souglis et al. [16]. However, one publication showed no significant effect of proprioceptive training on balance [23].

### Effects of PTM on performance

The effects of PTM on performance were examined in five studies [16, 23, 24, 27, 28]. Interventions were effective in improving explosive strength [24, 27], maximal strength [28], balance and flexibility [24], as well as agility [16]. Conversely, some studies showed no positive effects on explosive and maximal strength [28], as well as balance and flexibility [23].

### Effects of PTM on injury reduction and prevention

The effects of PTM on injury reduction and prevention were investigated in five studies [23, 26, 28]. In three cases, the aforementioned type of training had a positive effect on injury reduction and prevention [24, 26], in the remaining two studies, proprioceptive training did not affect the reduction of overall injuries [23, 28].

## Discussion

The aim of this research was to gather relevant studies on the effects of proprioceptive training on motor abilities and injury reduction in female soccer players. The main findings of the study highlight a partial impact of PMT on motor abilities and injury reduction and prevention. The partial impact of PTM interventions includes improvements of 66% in explosive strength, as well as 50% in maximal strength, balance, and flexibility. Regarding the impact of PTM interventions on injury frequency, a partial reduction (60%) in injuries among female soccer players after the intervention was observed. Prior mentioned PTM with varying duration, intensity, and frequency resulted in overall improvements in female soccer players.

### Effects of PTM on performance

The effects of PTM on performance were presented in five studies [16, 23, 24, 27, 28]. In three studies, the influence of proprioceptive training on strength modalities was examined [24, 27, 28]. These studies analyzed the impact of PTM on explosive strength of the lower extremities. Two studies found that proprioception influenced explosive strength of the lower extremities as observed through jump performances [24, 27]. However, in the study by Rodriguez [28], significant positive effects on vertical jump between initial and final measurements were not achieved. This indicates a success rate of 66% for the influence of proprioception on explosive strength of the lower extremities in female soccer players. Our findings are consistent with those of Chappell et al. [29], who argue that neuromuscular training significantly improves sports performance in jump tests. It should be noted that there were no significant positive effects on explosive strength of the upper extremities, assessed based on medicine ball throws [24], which is somewhat expected as the focus of the training process was on the lower extremities of the soccer players. In the only study [28] where authors investigated maximal force of the lower extremities, improvements in quadriceps and hamstring muscle strength of the right leg were found after the intervention, while there was no significant difference in the same parameters for the left leg, indicating a success rate of 50% (2 out of 4 parameters) for proprioception on maximal force of the lower extremity muscles in female soccer players. In two studies [23, 24], the authors examined the impact of PTM on balance and flexibility. Knobloh et al. [24] found that the experimental group achieved significantly better results in balance and flexibility tests compared to the control group. Conversely, in the study by Soderman et al. [23], no significant positive effects were found in the mentioned motor abilities. It can be concluded that this program has a relative success rate (50%) on balance and flexibility. One study investigated the impact of PTM on agility, and it was concluded that the intervention group achieved better effects on agility than the control group.

Based on these findings, it can be concluded that proprioceptive training has a partial positive impact on motor performance in female soccer players. However, PTM alone may not be sufficient for the development of motor abilities, and it should be used in conjunction with other training methods. These findings should be interpreted with caution due to the small number of included studies. Except for explosive strength, which was the subject of three studies, other motor abilities were examined in only one or two studies, which is insufficient to draw precise conclusions.

### Effects of PTM on injury reduction and prevention

The impact of proprioception on the frequency of injuries in female soccer players was examined in 5 studies [23–26, 28]. Significant reduction in injuries after proprioceptive training was found in three studies [24–26]. Conversely, in the remaining two studies, proprioceptive training did not affect the reduction of overall injuries [23, 24, 26–28]. Here, it could be argued that there is a 60% success rate of the given PTM in reducing injuries among female soccer players. It should be noted that the reduction in overall injuries in these studies varied considerably, from 35% in the Soligard study [25] to 65% in the Kraemer study [26], up to 400% as reported in the Knobloch study [24].

Results in men’s soccer following “balance training”, show that the incidence of anterior cruciate ligament (ACL) injuries have been dramatically reduced [30]. Two studies classified injuries according to severity [23, 25]. In the Soligard study [25], a positive effect of PTM on reducing major injuries was found, with 50% fewer major injuries reported among female soccer players after the intervention. In contrast, in the Soderman study [23], there were significantly more major injuries after the intervention compared to the control group, while there was no difference in minor and moderate injuries between the groups. This is the only parameter in which PTM showed significantly negative effects, so the reasons for such findings should be investigated. However, there were significant reductions in a number of secondary outcome variables, including cessation of serious injuries, overuse injuries, and total injuries. Previous intervention trials have emphasized core stability, balance, and neuromuscular control, as well as hip control and knee alignment to reduce excessive knee valgus during static and dynamic movements [30–35].

By analyzing the nature of injuries, the authors reported that non-contact injuries accounted for 72–73% of total injuries, while 27–28% of injuries occurred due to a contact with opponents [23, 24]. Consistent with this division, Kraemer [26] reported that PTM reduced non-contact injuries by 63%, while there was no effect of the intervention on contact injuries. Regarding lower extremity muscle injuries, Kraemer [26] found a reduction in hamstring muscle injuries by 43% and gastrocnemius injuries by 38%. In ligaments and tendons, reductions were observed in patellar tendon injuries by 50%, Achilles tendon injuries by 90%, and knee strain injury rates by 95%, with only ankle sprain injury not showing a decrease in frequency after the intervention. The risk of hamstring muscle injury among professional soccer players was considerably higher in individuals with untreated strength imbalances than players with no imbalance in preseason [36]. However, it should be noted that ankle sprain is the most common injury in female soccer players [23], indicating the importance of this data. Additionally, Kraemer [26] also found a significant correlation between the hours spent on PTM training and the reduction of injuries, where players who spent more hours doing PTM had a lower injury frequency.

Based on the provided findings, it is noticeable that PTM can be an important tool in the prevention and reduction of injury frequency among female soccer players. This observation mainly relates to the reduction of non-contact injuries concerning the muscular and ligamentous apparatus.

The strengths of this systematic review include that it provided deep insight into proprioceptive training methods in various qualitative levels of female soccer players completely supported by the previous studies. Also, it can be noted that this study provides extension of existing body of knowledge especially in identification of possible relations of PTM and reduction of injuries.

One of the limitations of this particular study is the small number of research (7) included in the final analysis. However, it is important to keep in mind that the selection criteria were established to ensure that only relevant and high-quality papers were included in the analysis. Despite this limitation, the study can still provide valuable insights and contribute to the existing body of knowledge on the topic. It is also possible for future research to build upon the findings of this study and expand the sample size to include more papers that meet the selection criteria. In the following, we could conclude that there is a real need for a meta-analysis in this field, which will fill gaps in the literature and provide more specific answers to the questions about the aforementioned type of training.

## Conclusion

In conclusion, lower limb balance and strength are important in soccer and should be built and maintained through proper training methods. Proprioceptive training has shown to be effective in improving athletic performance, reducing muscle injuries, and improving neuromuscular coordination, proprioception, and balance capabilities. However, not all intervention programs have shown significant benefits, and results may vary depending on the type and duration of the training. Studies have emphasized the importance of balance, neuromuscular control, and proper knee alignment to reduce the frequency of injuries. Overall, proprioceptive training should be an essential part of sports training programs to prevent injuries and improve athletic performance.

## Data Availability

No datasets were generated or analysed during the current study.

## Abbreviations

ACL: Anterior cruciate ligament
BMI: Body mass index
CNS: Central nervous system
PEP: Prevent injury and Enhance Performance
PTM: Proprioceptive training methods
SEBT: Star Excursion Balance Test
VO2max: Maximal oxygen consumption

## References

[1] Kandel ER, Schwartz JH, Jessell TM, Schwartz J, Jessell T. Princ Neural Sci. 1991;533–47.

[2] Lephart SM, Pincivero DM, Giraido JL, Fu FH (1997). The role of proprioception in the management and rehabilitation of athletic injuries. Am J Sports Med.

[3] Postle K, Pak D, Smith TO (2012). Effectiveness of proprioceptive exercises for ankle ligament injury in adults: a systematic literature and meta-analysis. Man Ther.

[4] Raymond J, Nicholson LL, Hiller CE, Refshauge KM (2012). The effect of ankle taping or bracing on proprioception in functional ankle instability: a systematic review and meta-analysis. J Sci Med Sport.

[5] Gollhofer A (2003). Proprioceptive training: considerations for strength and power production. Strength Power Sport.

[6] Aman JE, Elangovan N, Yeh I-L, Konczak J (2015). The effectiveness of proprioceptive training for improving motor function: a systematic review. Front Hum Neurosci.

[7] Page P (2006). Sensorimotor training: a global approach for balance training. J Bodyw Mov Ther.

[8] Álvarez-Serrano C, Alfaro-Segovia J, Guzmán-Muñoz E, Alarcón-Rivera M (2023). Neuromuscular training in football: a Literature Review. J Nov Physiother Rehabil.

[9] Ramos AVS, do Carmo RAL, Egídio CC. The importance of functional training in reducing the incidence of LCA injuries in footbolysts. 2018.

[10] Mohammadi F (2007). Comparison of 3 preventive methods to reduce the recurrence of ankle inversion sprains in male soccer players. Am J Sports Med.

[11] Cruz-Diaz D, Lomas-Vega R, Osuna-Pérez MC, Contreras FH, Martínez-Amat A. Effects of 6 weeks of balance training on chronic ankle instability in athletes: a randomized controlled trial. Int J Sports Med. 2015:754–60. 10.1055/s-0034-1398645 25969966

[12] de Vasconcelos GS, Cini A, Sbruzzi G, Lima CS (2018). Effects of proprioceptive training on the incidence of ankle sprain in athletes: systematic review and meta-analysis. Clin Rehabil.

[13] Hübscher M, Zech A, Pfeifer K, Hänsel F, Vogt L, Banzer W (2010). Neuromuscular training for sports injury prevention: a systematic review. Med Sci Sport Exerc.

[14] Gioftsidou A, Malliou P, Pafis G, Beneka A, Tsapralis K, Sofokleous P (2012). Balance training programs for soccer injuries prevention. J Hum Sport Exerc.

[15] Ojeda ÁH, Sandoval DC, Barahona-Fuentes G (2019). Proprioceptive training methods as a tool for the prevention of injuries in football players: a systematic review. Arch Med Deport.

[16] Souglis AG, Travlos AK, Andronikos G (2023). The effect of proprioceptive training on technical soccer skills in female soccer. Int J Sports Sci Coach.

[17] Rethlefsen ML, Kirtley S, Waffenschmidt S, Ayala AP, Moher D, Page MJ (2021). PRISMA-S: an extension to the PRISMA statement for reporting literature searches in systematic reviews. Syst Rev.

[18] Page MJ, McKenzie JE, Bossuyt PM, Boutron I, Hoffmann TC, Mulrow CD et al. The PRISMA 2020 statement: an updated guideline for reporting systematic reviews. BMJ. 2021;372. 10.1136/bmj.n71 PMC8005924 33782057

[19] Nishikawa-Pacher A (2022). Research questions with PICO: a universal mnemonic. Publications.

[20] de Morton NA (2009). The PEDro scale is a valid measure of the methodological quality of clinical trials: a demographic study. Aust J Physiother.

[21] Prictor M, Hill S (2013). Cochrane Consumers and Communication Review Group: leading the field on health communication evidence. J Evid Based Med.

[22] Maher CG, Sherrington C, Herbert RD, Moseley AM, Elkins M (2003). Reliability of the PEDro scale for rating quality of randomized controlled trials. Phys Ther.

[23] SöDerman K, Werner S, Pietilä T, Engström B, Alfredson H (2000). Balance board training: prevention of traumatic injuries of the lower extremities in female soccer players? A prospective randomized intervention study. Knee surgery. Sport Traumatol Arthrosc.

[24] Knobloch K, Martin-Schmitt S, Gösling T, Jagodzinski M, Zeichen J, Krettek C (2005). Prospektives propriozeptions-und koordinationstraining zur verletzungsreduktion im professionellen frauenfußballsport. Sport Sport.

[25] Soligard T, Myklebust G, Steffen K, Holme I, Silvers H, Bizzini M et al. Comprehensive warm-up programme to prevent injuries in young female footballers: cluster randomised controlled trial. BMJ. 2008;337. 10.1136/bmj.a2469 PMC2600961 19066253

[26] Kraemer R, Knobloch K (2009). A soccer-specific balance training program for hamstring muscle and patellar and achilles tendon injuries: an intervention study in premier league female soccer. Am J Sports Med.

[27] Gidu DV (2016). Influence of proprioceptive training on the strenght of the lower limb in women soccer players. Mircea Cel Batran Nav Acad Sci Bull.

[28] Rodriguez C, Echegoyen S, Aoyama T (2018). The effects of Prevent Injury and enhance performance program in a female soccer team. J Sport Med Phys Fit.

[29] Chappell JD, Limpisvasti O (2008). Effect of a neuromuscular training program on the kinetics and kinematics of jumping tasks. Am J Sports Med.

[30] Caraffa A, Cerulli G, Projetti M, Aisa G, Rizzo A (1996). Prevention of anterior cruciate ligament injuries in soccer: a prospective controlled study of proprioceptive training. Knee surgery. Sport Traumatol Arthrosc.

[31] Mandelbaum BR, Silvers HJ, Watanabe DS, Knarr JF, Thomas SD, Griffin LY (2005). Effectiveness of a neuromuscular and proprioceptive training program in preventing anterior cruciate ligament injuries in female athletes: 2-year follow-up. Am J Sports Med.

[32] Olsen O-E, Myklebust G, Engebretsen L, Holme I, Bahr R (2005). Exercises to prevent lower limb injuries in youth sports: cluster randomised controlled trial. BMJ.

[33] Myklebust G, Engebretsen L, Brækken IH, Skjølberg A, Olsen O-E, Bahr R (2003). Prevention of anterior cruciate ligament injuries in female team handball players: a prospective intervention study over three seasons. Clin J Sport Med.

[34] Emery CA, Cassidy JD, Klassen TP, Rosychuk RJ, Rowe BH (2005). Effectiveness of a home-based balance-training program in reducing sports-related injuries among healthy adolescents: a cluster randomized controlled trial. CMAJ.

[35] Heidt RS, Sweeterman LM, Carlonas RL, Traub JA, Tekulve FX (2000). Avoidance of soccer injuries with preseason conditioning. Am J Sports Med.

[36] Croisier J-L, Ganteaume S, Binet J, Genty M, Ferret J-M (2008). Strength imbalances and prevention of hamstring injury in professional soccer players: a prospective study. Am J Sports Med.

